# Involving Societal Stakeholders in Dementia Risk Reduction: An Explorative Study

**DOI:** 10.1111/hex.70541

**Published:** 2026-01-20

**Authors:** Jolanda H. M. Dobbe, Ellen M. A. Smets, Esmee Kreuk, Simone Coppelmans, Lars Ramaker, Moniek Schröder, Diny E. Stekelenburg, Wiebe de Vries, W. M. Monique Verschuren, Kay Deckers, Frank J. Wolters, Leonie N. C. Visser

**Affiliations:** ^1^ Medical Psychology, Amsterdam UMC location AMC University of Amsterdam Amsterdam the Netherlands; ^2^ Alzheimer Center Amsterdam, Neurology, Vrije Universiteit Amsterdam Amsterdam UMC location VUmc Amsterdam the Netherlands; ^3^ Amsterdam Public Health, Quality of Care Amsterdam the Netherlands; ^4^ Amsterdam Public Health Ageing and later life Amsterdam the Netherlands; ^5^ Amsterdam Public Health Personalized Medicine Amsterdam the Netherlands; ^6^ Independent Public Partner the Netherlands; ^7^ Department of Society Sports and Culture Municipality of Haarlemmermeer Haarlemmermeer the Netherlands; ^8^ Ben Sajet Centrum Amsterdam the Netherlands; ^9^ Department of Health Care University of Applied Science Leiden Leiden the Netherlands; ^10^ Center for Prevention, Lifestyle and Health National Institute for Public Health and Environment (RIVM) Bilthoven the Netherlands; ^11^ Department of Global Public Health and Bioethics, Julius Center for Health Sciences and Primary Care, University Medical Center Utrecht Utrecht University Utrecht the Netherlands; ^12^ Alzheimer Centre Limburg, Department of Psychiatry and Neuropsychology, Mental Health and Neuroscience Research Institute (MHeNs) Maastricht University Maastricht the Netherlands; ^13^ Departments of Epidemiology and Radiology & Nuclear Medicine Erasmus MC University Medical Center Rotterdam the Netherlands; ^14^ Department of Bioethics and Health Humanities, Julius Center for Health Sciences and Primary Care, University Medical Center Utrecht Utrecht University Utrecht the Netherlands; ^15^ Division of Clinical Geriatrics, Center for Alzheimer Research, Department of Neurobiology, Care Sciences and Society Karolinska Institutet Stockholm Sweden

**Keywords:** dementia, participatory research, population‐level approaches, risk reduction, societal stakeholders

## Abstract

**Objectives:**

Optimal dementia risk reduction requires a combination of individual‐ and population‐level approaches. Societal stakeholders play a crucial role by raising awareness, supporting individual lifestyle change, and/or influencing certain risk factors through policy changes. This study aimed to identify relevant societal stakeholders for promoting dementia risk reduction, and explore perspectives regarding their role.

**Methods:**

We used a qualitative approach with participatory research elements (i.e., collaborating with stakeholders in the research). An advisory panel of citizens (*n* = 14) was installed to provide input on various study aspects (e.g., study design and interpretation of findings). Thereafter, data collection involved two phases: 1) identification of potentially relevant societal stakeholders (based on advisory panel discussions, a conference workshop, and online searches); and 2) exploration of perspectives of participants from selected stakeholder domains, through 18 interviews and one focus group (total *N* = 32). We analysed data using thematic analysis.

**Results:**

Phase 2 revealed that participants, such as religious leaders, labour service employees and board members of student associations, had limited knowledge and experienced little responsibility to act as a societal stakeholder in the context of dementia risk reduction. Rather, they called for policy and regulations to make dementia risk reduction efforts obligatory and a public priority. Participants recommended incorporating information on dementia and dementia risk in general health campaigns, rather than organising dementia‐specific campaigns, and stressed the need to stimulate dementia risk reduction early in life.

**Conclusions:**

Effective dementia risk reduction could benefit from increased stakeholder involvement, as well as imposed policy‐level risk reduction measures. Our findings also highlight the importance of including dementia in education and healthy lifestyle programmes from an early age. Future studies are needed to validate our findings on a larger scale, and among different stakeholders.

**Patient or Public Contribution:**

Citizens were involved in study conceptualisation and design, and in the interpretation, reporting and dissemination of findings.

## 1 Introduction

Dementia has a significant impact on individuals' health and well‐being, on relatives, and on society at large [1]. The number of individuals living with dementia is expected to triple by 2050 due to the ageing population and the lack of effective treatment [2]. Consequently, the World Health Organization identified dementia prevention (i.e., reducing the risk of developing dementia, and slowing down or stopping disease progression) as a research priority [3, 4, 5].

The Lancet standing Commission on dementia prevention, intervention and care identified 14 evidence‐based, potentially modifiable risk factors for dementia across the life course: limited education in early life, hearing impairment, high LDL cholesterol, depression, traumatic brain injury, physical inactivity, diabetes, smoking, hypertension, obesity, excessive alcohol consumption, social isolation, air pollution and visual loss [6]. In total, these risk factors theoretically account for about 45% of dementia cases globally. Some of these modifiable risk factors, like education or air pollution, cannot be mitigated by making changes on an individual level, but require broader adaptations, for example, in the living or work environment [7, 8]. The remaining risk factors could be altered by medical interventions and/or behavioural change [2]. For all these factors, effective modification is challenging [9, 10, 11, 12, 13, 14].

Initiatives striving for dementia risk reduction have predominantly focused on the individual level, i.e., interventions or campaigns aimed at supporting and stimulating individuals' intentional lifestyle change [15]. The effects of these individual‐level approaches on dementia incidence are limited, and mostly benefit those with higher health literacy since they require cognitive, social and material resources, which are not evenly distributed across society [16]. As such, health inequalities in society persist or might even increase. A combination of individual‐level and population‐level approaches may be more effective for risk reduction. Population‐level approaches seek to decrease risk for all citizens by encouraging unconscious behavioural change or by eliminating exposure to risk factors. For instance, legislation could support healthy choices, and the introduction of noise standards could ensure that employees are less exposed to noise. For such societal changes, a shift is warranted in laws and regulations, policies, social norms, priorities, attitudes, and standard practices. This shift could be driven by individual actions, e.g., advocating for and architecting change, modelling behaviour, and/or making decisions in favour of dementia risk reduction.

In addition to citizens modifying individual behaviour for their own health benefit, some citizens could thus play a role in dementia risk reduction as a societal stakeholder. Societal stakeholders in dementia prevention involve professionals, volunteers, or organisations, such as healthcare professionals, employers, industry/companies, policymakers and educational institutions, that could contribute somehow to lowering the overall risk of dementia in society [7]. Healthcare professionals may, for example, facilitate individuals in adopting healthier lifestyle choices by providing information about dementia risk factors and risk reduction [14]. Additionally, the government could take an active role in dementia risk reduction by changing policy and practice, for example, by increasing taxation of unhealthy food products [17]. Moreover, policymakers could increase the availability of playgrounds, sport venues, and community centres [18], and employers could improve workplace conditions [19]. Hence, societal stakeholders could be pivotal in dementia risk reduction.

Only a few published studies have investigated views of (potential) societal stakeholders on dementia risk reduction. An interview study in Germany among 10 older adults and 10 stakeholders from fields such as architecture, environmental sciences and health policy, specifically explored how urban environments could stimulate healthy lifestyle and dementia risk reduction [8]. Themes that emerged related to social participation and inclusion, the importance of proximate facilities and services, and stimulation of recreation and wellbeing. Another study conducted interviews with 14 policymakers in the United Kingdom to assess their views on dementia preventability and individual‐ and population‐level risk reduction strategies [20]. Although dementia was perceived as preventable, there were concerns regarding the degree of preventability. Despite these initial insights, we lack insight into the variation in opinions of professionals who are expected to be able to promote dementia risk reduction (in the Dutch context) and how they view their responsibility. Therefore, our study aims to identify relevant societal stakeholders and assess perspectives regarding their role when it comes to dementia risk reduction.

## 2 Materials and Methods

### 2.1 Design

In this qualitative study, we used participatory research elements [21]. Participatory research involves the active engagement of those affected by the issue under investigation, aiming to educate and to foster action or change, through conducting research that is actually considered important [21, 22, 23]. Because we advocate that change is needed to reduce the risk of dementia, both at an individual and population level, we included citizens in the conceptualisation and design of the study, the interpretation of findings, scientific writing and dissemination of results.

The study was part of the Dutch BIRD‐NL consortium, which aims to stimulate dementia risk reduction through generating knowledge on modifiable risk‐ and protective factors of dementia [24]. The study was exempted from formal approval by the ethics committee of the Amsterdam University Medical Center, according to the Dutch law (2023.0543). All participants provided informed consent for the use of their data.

### 2.2 Establishment of an Advisory Panel

**Figure 1:**
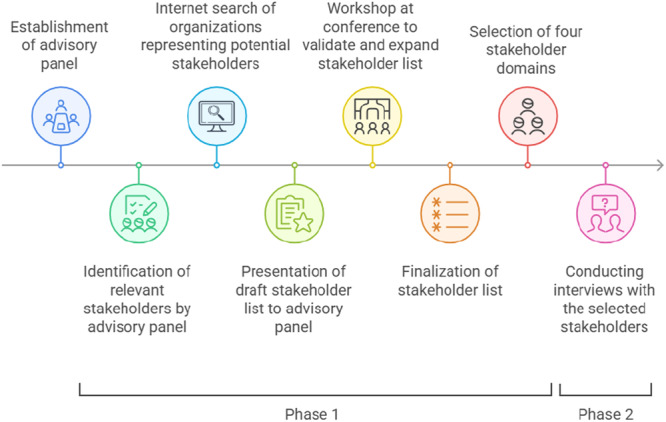
Research procedure. *Note.* Phase 1: Identification of relevant societal stakeholders; Phase 2: Exploration of perspectives in four domains of stakeholders. This figure was created using Napkin AI [25].

The research procedure is presented in Figure 1. First, an advisory panel was established, comprising citizens with a personal and/or professional interest in dementia and dementia risk reduction research. The advisory panel could provide input, based on their opinions, knowledge and expertise, in all phases of the current study, and on other studies conducted as part of the BIRD‐NL consortium. We used the following inclusion criteria: (1) proficiency in Dutch, both spoken and written; (2) age 18 or older; (3) commitment to participate for at least 1 year; and (4) ability to attend four meetings annually, preferably in person rather than online. Panel members were recruited through our own network, social media, the webpage of the Dutch Alzheimer's association (Alzheimer Nederland), and the newsletter of the Dutch Brain Foundation (Hersenstichting). Recruitment materials included an informational brochure and a video about the advisory panel. All individuals who expressed an interest (*N* = 37) were invited to join an informational online meeting. Of the 28 individuals who still expressed an interest afterwards, 16 were selected as a panel member based on their motivation, expertise, and demographic characteristics (i.e., age, gender, education, profession), aiming for a heterogenous group. The first meeting was held in October 2023, followed by quarterly meetings afterwards. Each meeting lasted approximately 2 h. If desired, members received a 25‐euro voucher per meeting attended, and reimbursement for travel expenses. Members provided written consent for the audio recording of meetings and the use of their input as data for this paper and potentially other publications.

### 2.3 Data Collection

After establishing the advisory panel, data were collected in two phases (see Figure 1).

#### 2.3.1 Phase 1: Identification of Relevant Societal Stakeholders

In Phase 1, we aimed to identify relevant societal stakeholders for dementia risk reduction. In an advisory panel session, our panel members brainstormed about potential stakeholders first in small groups and then plenary to generate a long list of stakeholders. Afterwards, JD and EK searched the internet for specific organisations in the Netherlands that might represent these stakeholders, which were added to the list. Thereafter, the list was presented to the advisory panel for validation and additions. To further expand and validate the list, we organised a workshop during the Dutch national dementia conference 2024, attended by people with dementia and their informal caregivers, healthcare professionals, researchers, educators, and policymakers. The workshop attendees were asked to provide input on potential stakeholders when aiming for dementia risk reduction, with the use of MentiMeter software. We specifically asked them who they considered as key persons because of their direct interaction with or influence on citizens at risk. The collected data were incorporated in the list, which was then finalised (see Appendix 1). From the final list, we selected four stakeholder domains for further exploration of perspectives in Phase 2, based on prioritisations made by the advisory panel. Healthcare professionals were not selected as a stakeholder domain, because these are specifically targeted in other ongoing research in the Netherlands.

#### 2.3.2 Phase 2: Exploration of Perspectives Within Four Stakeholder Domains

In Phase 2, interviews and focus group sessions were held between March 2024 and January 2025 with citizens recruited from selected stakeholder domains, aged > 18 years old and with sufficient fluency in Dutch. Participants were recruited through the networks of the authors, the consortium and the advisory panel members, by directly reaching out to specific organisations, and through snowballing. We strived for heterogeneity in age, gender and expertise, and used data saturation as a stopping criterion, i.e., that no new topics were introduced in two consecutive sessions. We conducted one focus group because there were multiple interested and relevant stakeholders from the same organisation. In the other cases, we conducted 2‐on‐1 or 1‐on‐1 interviews. The focus group and interviews aimed to determine participants': (1) knowledge of dementia risk and protective factors; (2) current ways of implementing dementia prevention (without specifying how we define ‘prevention’); (3) perspectives on their role in facilitating dementia risk reduction; and (4) ideas on how to increase people's awareness and/or motivation for dementia risk reduction. A semi‐structured topic guide was used (see Appendix 2), which was slightly adapted depending on the context of the participant(s). The interviews and focus groups were either conducted online or at the participants' workplace, were audio recorded, and ranged from 20 to 80 min. Data on sociodemographic characteristics (i.e., age, gender, educational level, work experience) were collected verbally. A summary of the focus group or interview was sent to participants afterwards for review, with only minimal feedback received.

### 2.4 Analyses

Descriptive statistics in SPSS‐statistics software version 28 were used to report sample characteristics [26]. For phase 2, thematic analysis was performed, aiming to identify patterns of meaning across the data, following six steps [27]. First, two researchers (JD (health scientist, junior researcher) and ES (medical psychologist, senior researcher)) listened to the audio recordings of the focus group and interviews, and independently wrote a summary for each session. These summaries were compared, discussed and combined to generate one comprehensive summary per session. Second, JD and ES both generated initial codes from the summaries using MaxQDA 2024 Software [28]. Third, based on the initial codes, themes were formulated independently by both researchers. Fourth, JD and ES compared their themes in a consensus meeting to produce a final list of themes. Fifth, names and specific descriptions were created for each theme. Sixth, a first draft of the results section was written, whereby illustrative quotes were selected from the audio‐recordings by JD. All authors reviewed these results in an iterative process, leading to the current manuscript. The advisory panel was also included in the analysis process, i.e., five panel members read the summaries and noted their thoughts and questions, and the initial themes were shared with the whole advisory panel to further shape them. In addition, the five panel members actively participated as co‐authors of this manuscript by critically reviewing the themes and manuscript as a whole. The Consolidated criteria for reporting qualitative studies (COREQ) were followed (see Appendix 3) [29].

### 2.5 Reflexivity

As researchers, we were aware that our professional backgrounds and personal perspective could shape the way we approached this study. The female researcher JD, with a background in health sciences and nutrition, conducted all interviews. She has been working as a nurse in an elderly care home, with people with dementia. This firsthand experience with dementia, and the particular attention to healthy lifestyle and dementia in her education, may have influenced how she interacted with participants and interpreted their views on dementia risk reduction. Throughout the interview process, she made field notes to capture initial impressions and reflections, and she shared written summaries with participants to ensure that their perspectives were accurately represented. Additionally, JD did not have a professional or other type of relationship with the participants, and she did not actively mention her professional background and research interests during the interview. The interview did start with an explanation of the study, and why it was deemed important. This could have steered participants' views. The data were independently coded by JD and ES, a female professor in medical psychology with a special interest in medical communication and education, in different health contexts. During consensus meetings, we reflected on how our disciplinary backgrounds informed codes and themes that emerged.

## 3 Results

### 3.1 The Advisory Panel

Initially, the advisory panel encompassed 16 members. Within the first year, two individuals dropped out because of time constraints and other obligations. The characteristics of the remaining 14 members are displayed in Table 1. All members had Dutch ancestry, except one individual with a second‐generation migration background, and they were mainly highly educated. In Table 2, five panel members shared their experiences, including reasons for signing up, contributions, and what participation brought them.

**Table 1:** Demographic characteristics of advisory panel.

Characteristics	Total (*n* = 14)
Age, *n* (%)	
20–35 years	5 (36)
36–50 years	1 (7)
51–65 years	3 (21)
> 65 years	5 (36)
Female, *n* (%)	9 (64)
Ancestry, Dutch, *n* (%)	13 (93)
Marital status, *n* (%)	
Married/registered partnership	6 (43)
Living together	3 (21)
Unmarried, never been married	5 (36)
Education, median (IQR)^a^	7 (6–7)
Working situation, *n* (%)^b^	
Paid job	8 (57)
Retired	5 (36)
Student	1 (7)
Years of experience in current or last job, *n* (%)	
< 1 year	0 (0)
1–5 years	7 (50)
6–10 years	2 (14)
11–20 years	2 (14)
> 20 years	3 (21)
Knows someone with dementia, *n* (%)	12 (86)
Care(d) for someone with dementia, *n* (%)	3 (21)
Functional health literacy, mean ± SD^c^	3.5 ± 0.6

Abbreviation: IQR, interquartile range.

^a^ Education is rated using the Dutch Verhage system, ranging from 1 to 7 [30].

^b^ The advisory panel members had the following occupations: teacher (nursing; nutrition and dietetics), graduation counsellor, primary education director, social support policymaker, elderly welfare advisor, engineering firm director, consultant, elderly care manager, cultural festivals project officer, occupational expert, naturopathic aromatherapist, programme officer public policy, affairs and communication, and student in Brain and Cognition in Society.

^c^ Functional health literacy is measured with the Dutch functional, communicative and critical health literacy scale [31]. The scale ranges from 1 to 4, with 1 representing low health literacy and 4 high health literacy.

**Table 2:** Exemplary experiences of advisory panel members.

Expertise	Experience as a panel member
Policy advisor elderly welfare at a municipality level (female, 29 years)	I joined the advisory panel with the aim to use my practical knowledge as a municipal policy advisor to enhance dementia prevention research. I believed that my insights could improve the study's relevance for stakeholders like municipalities. I enjoy being part of the advisory panel not only because I can contribute by giving advice but also because I gain invaluable knowledge about dementia research and prevention. The obtained knowledge directly informs my work as a policy advisor. Furthermore, I have had the pleasure to engage with interesting people and gain diverse perspectives on the topics we discuss. Joining the advisory panel has been an enriching and insightful experience.
Master student Brain and Cognition in Society (female, 24 years)	As a former psychobiology student, I learned a lot about the brain, from molecule to psyche, including dementia. Personally, I have seen firsthand how deeply dementia affects individuals and their families, as my grandmother lived with dementia for over a decade. As part of the BIRD‐NL advisory panel, I participate in brainstorming sessions, forming opinions and advice on proposed questions and topics. I love how we, as a diverse group, work together, combining our efforts, expertise, and experiences to work toward a common goal. The advisory panel meetings have helped me discover my passion for using my knowledge to address societally relevant challenges. This also guided my Master's choice. I am always thrilled to apply my skills and knowledge in favour of the advisory panel. I am really thankful to be part of the advisory panel, as this project has been a very positive and inspiring experience.
Policymaker social support law (former researcher informal care; male, 29 years)	I joined the advisory panel because of my knowledge in the social domain and the research I have done on the subject ‘meaningful activities for people with dementia’. I find my participation in the advisory panel very interesting because it gives me new insights in (new) research about the risk factors for dementia and how to address these risks within society. Where normally I focus on research about the quality of life for people with dementia and their caregivers, this gives me insights in the process and way how people can develop dementia on an individual‐level, but also on a population‐level. As an advisory panel, we have an active contribution in the search for the most important focus points and the framework for the research. Also, we have the opportunity to give input and feedback on the topic lists for questionnaires and points of interest.
Former director of elderly care organisation, currently retired (female, 69 years)	As a retired director of a healthcare organisation with both intramural and extramural care, I have observed significant advancements in supporting the needs and well‐being of people with dementia. Participating in the advisory panel enabled me to share my experiences while gaining insights from others, from various backgrounds. This experience broadened my understanding, particularly highlighting the role of hearing loss—alongside unhealthy lifestyle habits—in dementia prevention and the need for improved public communication on this issue. The panel's knowledge, experiences and expertise facilitated a well‐founded response to the research question: ‘What do Dutch people know about dementia risk and protective factors, and what motivates them to reduce their risk?’ Being part of this panel has been pleasant and informative, especially due to the exchange of knowledge and experiences.
Former project employee in the cultural sector, currently retired (male, 68 years)	Shortly after my retirement, I came across an article of the Dutch Alzheimer's association discussing the BIRD‐NL project. This initiative drew my attention due to my interest in health and lifestyle, in terms of ‘cause and effect’, how people can take charge and mitigate or postpone the burden of disease. Participating in the advisory panel provided an opportunity to expand my knowledge in an unfamiliar field, around a theme of increasing societal urgency. I am particularly attentive to the potential role of the cultural sector, as a lack of social (and cultural) participation and connection constitute modifiable risk factors for dementia. Additionally, participation in this project allows for collaboration with other individuals who also have an interest in this subject, in a professional setting with substantial guidance. Together we brainstorm and exchange knowledge, aiming to contribute to the prevention of dementia.

*Note:* Advisory panel members varying in age, gender and type of work were asked to write about their experiences.

### 3.2 Selection of Relevant Stakeholder Domains

From the initial list of potentially relevant stakeholders (Appendix 1), four stakeholder domains were selected for further exploration. The first domain concerned the work environment, involving those who could (in)directly influence the physical space and culture of a company and their employees' working conditions. Second, we selected the domain living environment, comprising stakeholders who may influence citizens' living conditions or surroundings, thereby possibly influencing risk factors which are not directly affected by individual behaviour. This encompassed stakeholders such as policymakers or individuals with a political function. Third, key persons in the community were selected, involving groups or individuals who could connect citizens in the community with researchers and/or healthcare professionals, such as a sports coach or a religious leader. This domain was deemed important by the advisory panel since key persons could potentially reach populations that are otherwise less engaged in (research aimed at) public health interventions. Fourth, student organisations were included, as the advisory panel emphasised the importance of stimulating dementia risk reduction from an early age, and student organisations may influence health behaviour of their members, for example through organisational regulations or providing educational materials. This domain encompassed both study associations (primarily aiming to educate and transfer knowledge) and student associations (aiming to stimulate social contact between students).

**Figure 2:**
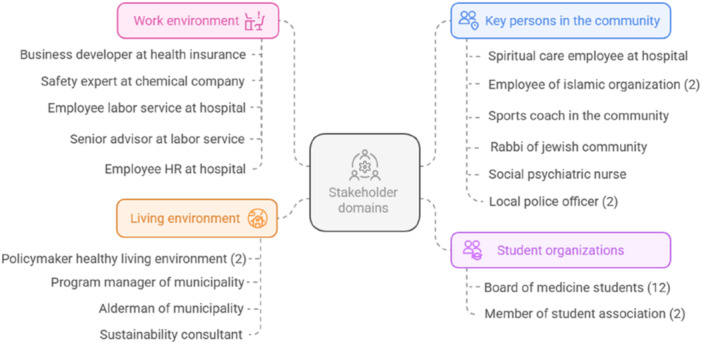
Overview of included stakeholders across four domains. *Note*. This figure was created using Napkin AI [25].

In total, 32 individual (potential) stakeholders were included as participants in Phase 2, of which 5 representing the work environment, 5 representing the living environment, 8 were considered key persons in the local community, and 14 were from student organisations. One focus group, three 2‐on‐1 interviews and 15 individual interviews were performed. An overview of the specific stakeholders that were included in each domain is shown in Figure 2, and Table 3 shows their demographic characteristics.

**Table 3:** Demographic characteristics of societal stakeholders.

Characteristics	Total (*n* = 32)
Age, *n* (%)	
20–35 years	15 (47)
36–50 years	8 (25)
51–65 years	7 (22)
> 65 years	2 (6)
Female, *n* (%)	21 (66)
Education, median (IQR) a	7 (6–7)
Years of experience in current job, *n* (%)	
< 1 year	4 (13)
1–5 years	19 (59)
6–10 years	4 (13)
11–20 years	4 (13)
> 20 years	1 (3)

Abbreviation: IQR, interquartile range.

^a^ Education is rated using the Dutch Verhage system, ranging from 1 to 7 [30].

### 3.3 Perspectives of (Potential) Societal Stakeholders

A total of 12 themes, divided over four categories, emerged from our analysis of the sessions with the potential societal stakeholders. These themes are further described below.

#### 3.3.1 Knowledge of Dementia Risk

##### 3.3.1.1 Participants' Understanding of ‘Dementia Prevention’

We noticed that a number of participants did not define ‘dementia prevention’ as reducing the risk of developing dementia. Instead, they emphasised the importance of timely detection of symptoms that may point to dementia, or taking into account people who will develop dementia in the future (e.g., in urban planning).

##### 3.3.1.2 Participants Questioned the (Degree of) Preventability of Dementia

Many participants questioned whether the risk of developing dementia was actually modifiable, or to what extent. Dementia was mainly perceived as a disease of old age, often genetically determined, and sometimes resulting from ‘just bad luck’. Nevertheless, several participants indicated lifestyle behaviours such as exercise, diet, and alcohol use as relevant for dementia risk, as well as social participation and mental activity. In contrast, dementia‐specific risk factors such as hearing loss, vision loss, limited education and air pollution were less known.

##### 3.3.1.3 Efforts to Promote General Health Rather Than Reducing Dementia Risk

Almost all participants indicated that in their (professional) role, they contributed to general health promotion only. They emphasised that this generic approach could also contribute to dementia risk reduction specifically, since many risk factors are not unique to dementia.

First of all, dementia is a disease, right? Of course you don't look at the living environment, via disease patterns. So you look at general aspects that are important for many things, right? Good for health. And now I don't know that much about dementia, but I have always understood that exercise is good, right? [….] Yes, but exercise is also good for cardiovascular diseases, diabetes, obesity, and everything. (policymaker healthy living environment).

(female, 51 years)

Participants offered various interventions and initiatives to promote general health and wellbeing of individuals. For instance, participants representing the work environment offered bicycle arrangements, healthy food during lunch, lifestyle checks, periodic medical examinations, and stress management programmes. These initiatives were mainly driven by reducing sick‐leave or stimulating employees' productivity.

#### 3.3.2 Perceived Responsibility for Dementia Risk Reduction

##### 3.3.2.1 Increasing Perceived Responsibility‐ and Attention to Health Promotion

Many participants felt a responsibility to promote general public health. Some participants already had a leading position in promoting public health and wanted to set a good example, while for others focusing on health promotion was rather new.

We are very active in this because we also want to be an example for other employers, so we also believe that we should be very healthy. So a lot of programmes are offered, we also have a multidisciplinary team ‘healthy working.’ (business developer at health insurance).

(female, 54 years)

The perceived responsibility for health promotion aligns with participants observing a societal development in which physical health is considered increasingly important. To illustrate, student organisation representatives indicated that attention to lifestyle is becoming more prominent in medical education and in their own daily living. Additionally, participants mentioned that young people find it increasingly important to make an impact with their work, for instance by focusing on health‐related issues. Similarly, participants representing the work environment indicated that employees encourage their organisational board to take collective action regarding health promotion of employees. They also mentioned that because of the rising age of retirement, sustained health and vitality of employees is becoming increasingly important and warrants attention to memory complaints at the workplace.

##### 3.3.2.2 Lack of Perceived Responsibility for Dementia Risk Reduction and Views on Who Should Be Responsible

With regard to dementia risk reduction specifically, participants rarely indicated to feel responsible to act as a societal stakeholder. Except for medical students, none of the participants saw dementia risk reduction as one of their tasks/duties or felt that they were the right person to educate others about dementia risk.

The police are not going to play a role in that anyway, because it is not primarily our job, so they will never do that… Because this is how they view it. Look, we do a lot of mental health work, of course. What I said, I often come across people who exhibit confused behaviour, but primarily this is not our job, […] Because it's not a priority, I think they're just saying very simply, yeah, that's not what police work should be like. (local police officer).

(male, 41 years)

Although medical students felt responsible for informing patients about risk‐ and protective factors of dementia, they also emphasised that it may be more effective to focus on how to improve one's lifestyle in general. Consequently, in various sessions the question arose who should take responsibility for dementia risk reduction. Various participants pointed to the role of politics, the government and policymakers. They argued that policy and compulsory regulations are needed so that dementia risk reduction becomes a public priority. Besides, they suggested the government could provide (financial) resources to make preventive measures more (equally) accessible. Additionally, participants indicated that broader societal adaptations were required, as some risk factors of dementia such as air‐ and noise pollution are difficult for the individual to influence. These broader adaptations could be induced by the government. Also, participants advocated for promoting healthy choices, for example by increasing the availability of healthier take‐away restaurants, and lowering prices for healthy food products. Lastly, one participant mentioned that health insurers could play a role by investing preventively in healthy living, for example by expanding the basic insurance.

‘Well, if you ask me, but I think that's the case with a lot of healthcare issues. The government simply needs to focus more on prevention, and prevention is just not considered important. Dietetics is not even reimbursed, you know, those are the kinds of things they abolish first.’ (social psychiatric nurse).

(female, 36 years)

I think that there are certain points that, as a government, as a policymaker, you just have to ensure that it is simply not possible for people to be exposed to it. Because you cannot leave everything to the individual. […] You can't force someone to go to the gym, […] but you can really stimulate them enough and create awareness, and make it easy for them, right? (board member of student organisation).

(female, 26 years)

However, some ethical concerns were raised as a consequence of shifting responsibilities from the individual towards the government. On the one hand, participants indicated that it is important to take good care of citizens from a moral perspective, and that the government could fulfil this role. On the other hand, doing so may limit individuals' freedom of choice.

#### 3.3.3 Recommendations for Knowledge Dissemination

##### 3.3.3.1 Importance of the Messenger in Providing Information about Dementia Risk Reduction

Several recommendations were proposed regarding by whom information on dementia risk reduction could be communicated. Some participants suggested individuals may be more likely to accept information from experts or authorities such as their doctor, scientists or the Dutch Alzheimer's Association, or from individuals with lived experience, because they can make the message more appealing. One participant mentioned that when the message is conveyed by the government, this might actually work counterproductive for some groups:

There is also just little trust in institutions, that's a very important one. It is simply due to the things they have experienced […]. So they also have a lot of distrust. [.…] And the general attitude of the government towards, in our case towards Islam and Muslims, is also openly and clearly negative. (employer of Islamic organisation).

(female, 36 years)

##### 3.3.3.2 Importance of Early Education

Regarding when knowledge should be conveyed, participants highlighted the importance of early, i.e. at a young age, and recurrent education, to embed attention to risk factors within the collective consciousness. In this way, healthy behaviour will be normalised.

I think the earlier, the younger you start, the better. That it becomes normal and that it's part of the upbringing. So I think, start with a game in primary school, then continue in secondary school […] And I think, if you then keep bringing that back in different age phases. And that you go into it a little deeper each time. But the basis starts early, then it is seen as normal. Then it is not something new, a must, like ‘We have to do it’, because then you immediately get resistance. (board member of student organisation).

(female, 26 years)

##### 3.3.3.3 Participants Advocate for Clustering Diseases in General Health Campaigns

Most participants agreed that because of shared risk factors, health campaigns should integrate multiple diseases rather than focusing exclusively on dementia. Such an all‐encompassing strategy was considered to enhance motivation for behavioural change. Still, participants also emphasised the importance of providing education on dementia‐specific risk factors.

I think […], the more specific your knowledge is, the more targeted you can make your own choices. […] And I don't know if that works for everyone, but I do see it in my environment. That knowledge does lead to making more conscious choices. (safety expert at chemical company).

(male, 65 years)

##### 3.3.3.4 Importance of Tailored Interventions with Positive Language

Participants expressed the relevance of involving the target population in designing interventions. Offering a variety of health interventions was also considered important, to tailor to variation in individual needs. Additionally, participants mentioned interventions could be aimed at specific target populations by focusing on risk factors that have a greater relevance for the specific stakeholder (e.g., risk factors affecting the living environment for policymakers), and offering stakeholders guidance on how to tackle these risk factors. In terms of language use, some participants suggested that the message should be presented in a positive way, for example by emphasising how individuals can exert control over their lifestyle choices, and highlighting the health benefits of behavioural change. Participants also mentioned the importance of avoiding messages that create a sense of obligation, because this would potentially work counterproductively. Finally, they highlighted that information should be accessible, include humour, and should be presented in different ways, because some individuals may respond more positively to the concept of ‘brain health’ while others may be more engaged by the term ‘dementia’.

Those spiritual care factors, for example I see depression, social isolation [points to figure with risk factors]. So I think, well that could also have a protective effect if you pay attention to it. […] So that is perhaps a bit wrapped up in it, but you could still say that in a positive sense it is important that attention is paid to that. (employee spiritual care at hospital).

(female, 64 years)

#### 3.3.4 Perceived Factors Motivating and Hindering Dementia Risk Reduction Efforts

##### 3.3.4.1 Influence of Experience With Dementia and Behaviour of Peers

The interviews revealed that solely providing knowledge was considered insufficient to encourage people to change their behaviour. Several participants argued that personally knowing someone with dementia may motivate taking preventive measures, since it is likely to increase awareness of the consequences of the disease and a sense of urgency. Additionally, some participants indicated that, especially at a young age, healthy behaviour of peers may have a positive influence. However, this could also have a negative impact, for example when unhealthy behaviour is the social norm:

Yes, I think, if you order it [alcohol‐free drink] at the bar, people do look at you a bit like, huh? Or at least, […] Yes, the unwritten rule is that you order beer or something else. (member of student association).

(female, 25 years)

##### 3.3.4.2 Lack of Urgency, Visibility and Resources for Dementia Risk Reduction

Participants also acknowledged some hindering factors for dementia risk reduction, such as the lack of perceived urgency. As previously mentioned, dementia is often associated with old age and thus with something that may occur in the future. As a consequence, motivating individuals to change their behaviour early in life is difficult. Students indicated that they often justify their unhealthy behaviour during their student days:

Those [students] all think, Oh yeah, it'll be fine, that's [hearing loss] a problem for later. […] Same with alcohol use, or smoking. Everyone looks at it like, ‘I'll only smoke for a few years and then I'll quit or, oh, I'm a student, so I'm allowed to drink. (board member of student association).

(female, 22 years)

In addition, some participants mentioned that individuals who would benefit most from health promotion interventions, such as socially isolated people of older age, are often not visible to healthcare workers and are not accessing these interventions. Similarly, participants recognised that the extent to which people have control over their own health behaviour partly depends on livelihood security (i.e., having sufficient income to meet basic needs). For instance, they mentioned the ability to buy healthy food and to participate in social activities. Participants indicated that only once requirements for livelihood security are met, individuals start to concern themselves with their health.

##### 3.3.4.3 Challenges Related to Investing in Health Promotion Initiatives

Among participants representing the living environment, the limited direct yield of prevention or health promotion was perceived as a barrier for dementia risk reduction. Investments are made by someone (e.g., project developers), but the return often goes to someone else (e.g., the individual, society). While the government has a responsibility to contribute to the health of the population, project developers do not feel this responsibility. Additionally, participants mentioned that the effect of health interventions is difficult to measure.

But actually make it measurable and provide insight into what the health effects are per measure or per project, yes, you can hardly do that. And when you do it based on existing methods and research, then that is sometimes almost demotivating. Then it is like, well, this contributes only a little. (policymaker at municipality).

(female, 36 years)

Participants indicated that due to limited resources and different interests, trade‐offs often have to be made, which mostly result in concessions on health promotion.

## 4 Discussion

In this study, we identified a broad variety of potential societal stakeholders for dementia risk reduction. However, potentially relevant stakeholders representing the work and/or living environment, and key persons in the community, often did not see a role for themselves when it comes to specifically stimulating dementia risk reduction. Instead, they did see a role in promoting general health. Medical students were the exception, indicating to feel responsible for informing patients about risk‐ and protective factors for dementia. Participants generally emphasised that the government and policymakers should have a greater role in dementia risk reduction.

Participants were doubtful about the modifiability of dementia risk, and their knowledge about risk and protective factors was relatively low, particularly about non‐lifestyle factors (such as social factors or air pollution). An interview study among 14 policymakers confirms these findings, with policymakers mainly identifying factors related to lifestyle as risk factors for dementia, and less frequently indicating dementia‐specific risk factors like social isolation. In addition, some policymakers had doubts about prevention being possible [20]. Similar findings were observed among healthcare professionals and students [14, 32, 33, 34, 35, 36, 37, 38, 39, 40, 41, 42, 43, 44, 45]. Moreover, two studies with community leaders (e.g. religious leaders and local council leaders) demonstrated awareness of lifestyle‐related risk factors like diet, alcohol consumption and hypertension, but also misconceptions such as evil spirits [46, 47]. As the evidence illustrates, knowledge of risk and protective factors among various populations who could serve as societal stakeholders in dementia risk reduction is suboptimal. However, we argue that having accurate and adequate knowledge is a prerequisite for educating others about dementia risk reduction, or to facilitate change in other ways, like changing policy and practice.

Perceived responsibility for dementia risk reduction in our study was low. Professionals often referred to the responsibility of higher‐order policymakers, particularly the government, in this regard. This tendency to attribute responsibility to the government is also observed in the domain of smoking cessation, a known risk factor for dementia. A Dutch interview study found that physicians indicated that the government should take responsibility to prevent individuals from smoking, although they viewed smoking cessation also to be a mutual responsibility of healthcare professionals and patients [48]. These physicians highlighted the insufficient priority assigned to smoking cessation support by the government, which resonates with the opinion of participants in our study with regard to dementia risk reduction. Nevertheless, regarding smoking cessation, global tobacco control policies such as taxes, smoking bans and health warnings have been introduced over the last decade, thereby increasing the responsibility of the government [49]. For other risk factors of dementia, such an increase in governmental responsibility has not yet taken place, although dementia prevention has been a key part of the Dutch governmental National Dementia Strategy since 2021 [50]. A scoping review examining available dementia risk reduction policies and strategies in England, argued that translating research into policy is a slow and complex process, which may explain why dementia risk reduction is not yet integrated in policy [51]. Moreover, it is often not acknowledged that systemic level changes to promote dementia risk reduction result in reducing healthcare costs of dementia, in part due to difficulty in measuring intervention effects [20, 52]. This asks for a more altruistic or social value viewpoint, wherein health is not solely evaluated in economic terms, and societal responsibility to ensure wellbeing of all citizens is emphasised.

With regard to how knowledge about dementia risk reduction should be disseminated, most professionals suggested integrating dementia in health campaigns for other conditions by focusing on shared risk factors, while some highlighted the importance of providing education on dementia‐specific risk factors. These different views are also observed in the literature. For example, Dutch primary care providers valued integration because it facilitates in cardiovascular risk management [53]. Similarly, policymakers in the United Kingdom questioned the added value of dementia‐specific campaigns because many risk factors are already tackled in other public health policies [20]. Both the primary care providers and policymakers also indicated that the emotional connection to dementia may act as additional motivation for behavioural‐ or policy change [20, 53], as previously observed for other chronic conditions [54]. Taken together, this suggests a two‐track policy for dementia risk reduction, integrating dementia in general health campaigns of diseases with shared risk factors, while organising targeted campaigns to address dementia‐specific risk factors such as depression, social isolation and hearing loss. We advocate for interventions aimed at specific stakeholders, as our participants indicated that stakeholders would only be engaged when a risk factor bears relevance to their specific situation and when they have some direct influence on a risk factor.

Professionals highlighted the importance of education at an early age about risk and protective factors for dementia. At the same time, they acknowledged the lack of perceived urgency or motivation to change behaviour in favour of dementia risk reduction at a young age. The perspective of stimulating dementia risk reduction from an early age is supported by other research [20, 52, 55, 56]. For example, an expert group on brain health argued that young adults are highly exposed to risk factors, and unhealthy behaviour during adulthood can have long‐term consequences on health [55]. In line, novel tools are being developed to educate children about brain health, by using animated videos or exercises [56]. One way to motivate individuals to change their behaviour early in life may be by highlighting the short‐term benefits of risk reduction. For example, for employers, short‐term benefits of promoting their employees' health could be less sick‐leave due to burn‐out, increased productivity, or increased employee commitment or satisfaction [57, 58, 59]. Another way to stimulate risk reduction in early life could be to emphasise that dementia can already develop at a young age, thereby changing the perception that dementia is something only occurring in the distant future. However, such an approach comes with ethical considerations [60], like whether it is allowed to possibly instill fear in order to achieve dementia risk reduction. Finally, educating the younger generation about dementia risk reduction may enable them to serve as a bridge to communicate relevant information to older generations. Yet, optimal prevention from a young age onwards likely also requires population‐level interventions to ensure for example a healthy food environment, playgrounds for physical activity, and community places.

Among the strengths of our explorative study is the participatory approach that we applied, involving stakeholders already from the start of the research. There has been growing attention to public involvement in health‐ and dementia care research [22, 23, 61, 62], since addressing public needs in research can enhance the uptake of findings and motivate to take action, and public involvement has been shown to improve health outcomes, knowledge, self‐worth, and quality of life for those involved in the study [21, 22, 23]. However, as the included stakeholders mainly concerned individuals without a migration background, with high levels of education, who knew someone with dementia, our findings may not be representative for the Dutch population. These stakeholders may already have increased motivation to adopt a healthy lifestyle, and may have come up with potential societal stakeholders of which they thought could have an influence on themselves or their direct environments. Thereby other important stakeholders could have been neglected. Moreover, in phase 2 of our study we might have particularly attracted individuals with a high(er) interest in dementia risk reduction and general health, thereby providing an optimistic view of participants' perceived importance of dementia risk reduction. For instance, participants highlighted the importance of prioritising health promotion through policy, but policymakers may only perceive this as important once they have personal experience with dementia or when they need support to improve their own health. Therefore, our recommendations should be interpreted with caution. Further research is needed to validate our findings on a larger scale, for example by means of a survey. Finally, the background of the interviewer (health sciences and nutrition) may have directed the topics that were discussed, although interview guides and protocols were used.

## 5 Conclusion

In this explorative study we selected four domains of societal stakeholders when it comes to dementia risk reduction, and assessed perspectives of domain representatives regarding their role in dementia risk reduction. The potential stakeholders that we included in our study felt little responsibility for contributing to dementia risk reduction specifically. Rather, they pointed to the role of other higher order stakeholders, such as the government, for policy‐level interventions. Nevertheless, participants underscored the importance of promoting the general health of their fellow citizens, and of integrating dementia risk reduction in existing public health campaigns. Future research is needed to test the effectiveness of such an approach. Furthermore, validation of our findings with regard to societal responsibility on a larger scale, including stakeholders from other domains, would be of added value.

## Author Contributions

**Jolanda H. M. Dobbe:** conceptualisation, methodology, investigation, formal analysis, visualisation, writing – original draft, project administration. **Ellen M. A. Smets:** conceptualisation, methodology, formal analysis, writing – review and editing, supervision, project administration, funding acquisition. **Esmee Kreuk:** conceptualisation, methodology, investigation, writing – review and editing. **Simone Coppelmans:** conceptualisation, formal analysis, writing – review and editing. **Lars Ramaker:** conceptualisation, formal analysis, writing – review and editing. **Moniek Schröder:** conceptualisation, formal analysis, writing – review and editing. **Diny E. Stekelenburg:** conceptualisation, formal analysis, writing – review and editing. **Wiebe de Vries:** conceptualisation, formal analysis, writing – review and editing. **Frank J. Wolters:** conceptualisation, writing – review and editing, funding acquisition. **Leonie N. C. Visser:** conceptualisation, methodology, formal analysis, writing – review and editing, supervision. **W. M. Monique Verschuren:** writing – review and editing. **Kay Deckers:** writing – review and editing.

## Ethics Statement

The study was exempted from formal approval by the ethics committee of the Amsterdam University Medical Center, according to the Dutch law (2023.0543). All participants provided informed consent for the use of their data.

## Conflicts of Interest

The authors declare no conflicts of interest.

## Supporting information

Supplementary material_Societal stakeholder involvement dementia risk reduction_not anonymized.docx.

## Data Availability

The data that support the findings of this study are available from the corresponding author upon reasonable request.
