# Supplementary material for: Involving Societal Stakeholders in Dementia Risk Reduction: An Explorative Study
Source: Health Expect. 2026 Jan 20;29(1):e70541. doi: 10.1111/hex.70541 (PMC12820418; doi:10.1111/hex.70541)
Supplement: Supplementary file 1 — Supplementary material_Societal stakeholder involvement dementia risk reduction_not anonymized.docx. [file HEX-29-e70541-s001.docx]

# Supplemental material

## Appendix 1: Longlist of societal stakeholders

- Teachers at primary and secondary education
- Deans
- LAKS: representative of Dutch secondary school students
- National Consultation on Primary Education Teacher Training (LOBO)
- Professional association of Vocational Education Training
- V&VN Trainers: aimed at trainers of nurses and carers
- Dutch Association for Educational Personnel (NVOP)
- Federation of Educational Organizations (FVOV)
- Association of Universities of Applied Sciences
- Primary and secondary education council
- National training consultation for nurses (LOOV)
- Organization ‘De geneeskundestudent’ (The medical student)
- Intercity Student Consultation (ISO): national organization representing the interests of students.
- National Student Union (LSVb)
- Youth Organization Vocational Education
- National Chamber of Associations
- Salus; study association for health sciences at the Vrije Universiteit Amsterdam.
- General practitioner
- Practice assistant (POH)
- Health insurances
- Physiotherapist
- Dietician
- Case manager
- Community nurse
- Informal caregivers
- Care coordinators
- Welfare workers/organizations
- Elderly advisors
- Day care workers
- Domestic helper
- Mental healthcare workers
- National healthcare organizations;
  - National institute for public health and environment (RIVM)
  - Association of GGD’s (Regional Public Health Services) and GHOR-(Regional Medical Emergency Preparedness and Planning) offices in the Netherlands (GGD GHOR Nederland)
  - Dutch healthcare institute
- Environmental Planning Agency
- Urban planners
- Architects
- Engineers
- Policy makers
  - Association of Dutch Municipalities
- Real estate participation broker
- Employers
- Dutch Association for Occupational and Occupational Medicine (NVAB)
- Staff associations
- Pension funds
- Prevention workers
- Churches
- Mosques
  - Imam
  - Ulu Mosque in Utrecht (The Netherlands)
- Protestant National Service Center
- Protestant Church in the Netherlands (KPN)
- The Roman Catholic Church in the Netherlands (RCK)
- Ahmadiyya Muslim community
- Association of Dutch Preachers
- Platform Islamic Organizations Rijnmond Foundation (SPIOR)
- Catholic interest group for seniors aged 50 and older (KBO)
- Local police officers
- Municipalities:
  - Alderman for health care
  - City council
  - Social support law- employees
  - Healthcare team
- Community centers
- Social web
- Social worker
- Social district team
- Youth worker
- Debt assistance
- Centre for domestic violence
- Voluntary workers
- Shops
- Sports:
  - Sports club > coaches
  - Associations like Royal Dutch Hockey Association (KNHB), Royal Dutch Soccer Association (KNVB) and Dutch Boxing Association (NBB)
  - Sports and municipalities association
- Library employees
- Culture center employees
- Hobby clubs
- Choir
- Music festival employees
- Game makers
- Influencers
- Famous Dutch people
- Alzheimer Nederland – ‘samen dementievriendelijk’ (together dementia-friendly) initiative.
- Dutch Brain Foundation
- Alzheimer café

## Appendix 2: Interview topic guide (example of living environment).

**Introduction**

- Explain aim and structure of the interview;
  - Background information about BIRD-NL consortium and included work packages
- Introduction round;
  - Age
  - Study background
  - Current job function
  - Years of working experience
  -
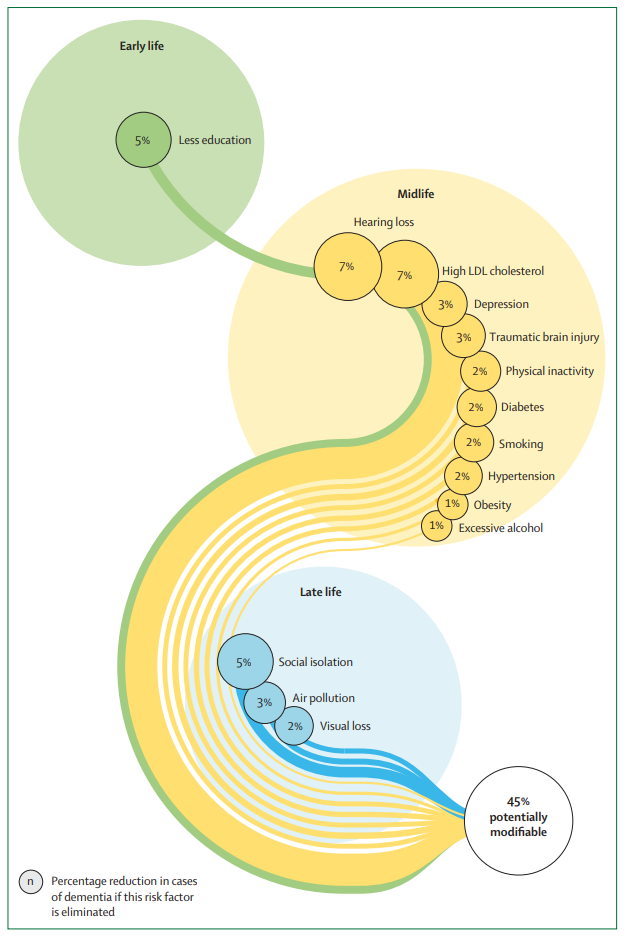
Work activities

**Part 1: Knowledge about risk- and protective factors**

- What do you think are risk factors for dementia?

*Show Figure of Livingston paper; shows 14 risk factors for dementia over the life course, and indicates at what point in life these risk factors play the greatest role. Together, these modifiable factors explain 45% of the total dementia risk. This means that if you were to address all these factors, 45% of dementia cases could potentially be prevented.*

- Which of these risk factors did you know of and which didn't?
- Are there risk factors that you thought would play a greater or lesser role?
- Do you miss any risk factors?
- Do you have any questions or thoughts based on this figure?

**Part 2: dementia prevention in the organization**

- Do you currently take public health and dementia prevention into account when making plans for the living environment?

If yes:

- - How do you take it into account?
  - Are conscious choices made in planning to promote public health, and how?
  - Any policies/guidelines being followed?
  - What sources of knowledge do you rely on?
  - Is prevention a priority in your organization?
  - Do you also collaborate with other organizations (municipality, project developers, the government etc.)?

If no:

- - Why not?
- Do you consider yourself/your organization as a societal stakeholder in dementia prevention? Why/why not?

If not:

- - Who should be responsible?
- What other societal stakeholders do you consider important?

**Part 3: Challenges and future vision**

- Which challenges do you encounter with regard to implementing public health/prevention initiatives?
- How do you see the future when it comes to a healthy living environment and prevention of dementia?
  - What changes are needed?
- How would you want to take dementia prevention into account?
- Which specific risk factors could you tackle?
- Which persons/organizations are important to have on board?
- Is it also important that the new generation of employees in this field (i.e. planners and/or spatial designers of the future) pay more attention to this subject?
  - If yes, should education focus more on this subject?
  - Where should we pay attention to? What should we tell people about this topic?

**Part 4: Increasing awareness about the importance of dementia prevention**

- What could we as researchers do to increase awareness among spatial designers about the importance of risk reduction/healthy living environment?
  - What message should we convey?
  - What arguments could we use to address them? So what would be a reason for them to get motivated to take dementia prevention into account?
  - Through which channels can we best reach this group? (e.g., social media, flyers, campaigns, someone within the organization)

**Closure**

- Are there any other relevant parties to talk with?
  - People/organizations that are not yet much involved with the subject?

## Appendix 3: COREQ checklist [29]

| **No** | **Item** | **Guide questions/descriptions** |
| --- | --- | --- |
| **Domain 1: Research team and reflexivity** | | |
| Personal characteristics | | |
| 1. | Interviewer/facilitator | Which author/s conducted the interview or focus group?  *JD. She was involved in data collection, analysis and interpretation of findings.*  *ES (Prof. Dr.) was involved in data analysis and interpretation of findings. LV (PhD) was included in the interpretation of findings.* |
| 2. | Credentials | What were the researcher's credentials? E.g. PhD, MD  *JD: MSc*  *ES: Prof. Dr.*  *LV: PhD* |
| 3. | Occupation | What was their occupation at the time of the study?  *JD: PhD candidate, Amsterdam UMC (department of Medical Psychology)* |
| 4. | Gender | Was the researcher male or female?  *JD: Female* |
| 5. | Experience and training | What experience or training did the researcher have?  *The interviewer followed courses on qualitative research during her bachelor’s degree and PhD trajectory. Additionally, she conducted various focus groups and interviews as part of different courses, her masters’ internship and research projects during her PhD trajectory.* |
| Relationship with participants | | |
| 6. | Relationship established | Was a relationship established prior to study commencement?  *The interviewer did not have a professional or other type of relationship with the study participants, and she did not actively mention her professional background and research interests during the interview.* |
| 7. | Participant knowledge of the interviewer | What did the participants know about the researcher? e*.g. personal goals, reasons for doing the research*  *The interview started with an explanation of the study, the overall research aim, and why the research was deemed important.* |
| 8. | Interviewer characteristics | What characteristics were reported about the interviewer/facilitator? e.g. *Bias, assumptions, reasons and interests in the research topic*  *The interviewer has a background in health sciences and nutrition. Additionally, she has been working as a nurse in an elderly care home, with people with dementia.* |
| **Domain 2: Study design** | | |
| Theoretical framework | | |
| 9. | Methodological orientation and Theory | What methodological orientation was stated to underpin the study? *e.g. grounded theory, discourse analysis, ethnography, phenomenology, content analysis*  *Thematic analysis was used.* |
| Participant selection | | |
| 10. | Sampling | How were participants selected? *e.g. purposive, convenience, consecutive, snowball*  *Participants were selected through purposeful sampling (e.g., the networks of the authors, the consortium, the advisory panel members and by directly reaching out to specific organizations), and snowballing.* |
| 11. | Method of approach | How were participants approached? e*.g. face-to-face, telephone, mail, email*  *Through email.* |
| 12. | Sample size | How many participants were in the study?  *14 advisory panel members, 32 focus group- and interview participants.* |
| 13. | Non-participation | How many people refused to participate or dropped out? Reasons?  *2 members of the advisory panel dropped out because of limited availability, overlapping responsibilities and other obligations.*  *Multiple individuals did not respond to our invitational email for a focus group or interview, but these were not recorded. The individuals who did respond to our email either wanted to participate themselves, or referred us to someone who eventually participated.* |
| Setting | | |
| 14. | Setting of data collection | Where was the data collected? e*.g. home, clinic, workplace*  *Online (Microsoft teams) or at the participants’ workplace.* |
| 15. | Presence of non-participants | Was anyone else present besides the participants and researchers?  *No* |
| 16. | Description of sample | What are the important characteristics of the sample? *e.g. demographic data, date*  *The participants of the focus groups and interviews (n=32) were individuals involved in one of the following four domains: 1) student organizations, 2) living environment, 3) work environment, 4) key persons in the community. The greatest part (46.9%) was aged 20 – 35 years, 25% was 35 – 50 years, 21.9% aged 50-65 years and the remaining (6.3%) had an age of > 65 years. 65.5% of the participants were female, they were highly educated and most often had 1-5 years of working experience in their current or last (in case of retired participants) job (59.4%).* |
| Data collection | | |
| 17. | Interview guide | Were questions, prompts, guides provided by the authors? Was it pilot tested?  *We made use of a topic guide, included as a supplemental file, providing guidance on the interview. This topic guide was not pilot tested.* |
| 18. | Repeat interviews | Were repeat interviews carried out? If yes, how many?  *No* |
| 19. | Audio/visual recording | Did the research use audio or visual recording to collect the data?  *Yes, we used audio-recording.* |
| 20. | Field notes | Were field notes made during and/or after the interview or focus group?  *Yes* |
| 21. | Duration | What was the duration of the interviews or focus group?  *The duration ranged from 20 to 80 minutes.* |
| 22. | Data saturation | Was data saturation discussed?  *Yes, we strived for heterogeneity in age, gender and expertise, and used data saturation as a stopping criterion, i.e., that no new topics were introduced.* |
| 23. | Transcripts returned | Were transcripts returned to participants for comment and/or correction?  *Transcripts were not returned, but summaries were returned to provide the opportunity to adjust and/or add information.* |
| **Domain 3: analysis and findings** | | |
| Data analysis | | |
| 24. | Number of data coders | How many data coders coded the data?  *Two* |
| 25. | Description of the coding tree | Did authors provide a description of the coding tree?  *No.* |
| 26. | Derivation of themes | Were themes identified in advance or derived from the data?  *Themes were derived from the data.* |
| 27. | Software | What software, if applicable, was used to manage the data?  *MaxQDA software.* |
| 28. | Participant checking | Did participants provide feedback on the findings?  *Focus group and interview participants provided feedback on the summaries that were created based on the audio-recording. They did not provide feedback on the manuscript. Five members of the advisory panel did provide feedback on the manuscript.* |
| Reporting | | |
| 29. | Quotations presented | Were participant quotations presented to illustrate the themes / findings? Was each quotation identified? e*.g. participant number*  *Participant quotations were presented, and identified by adding the profession of the participant.* |
| 30. | Data and findings consistent | Was there consistency between the data presented and the findings?  *The presented data aligned with the findings.* |
| 31. | Clarity of major themes | Were major themes clearly presented in the findings?  *Based on the analysis, four overarching categories emerged, including 12 themes, which were presented in the results section.* |
| 32. | Clarity of minor themes | Is there a description of diverse cases or discussion of minor themes?  *We tried to balance the discussion of common and minor themes, as well as providing space for different opinions.* |
